# miR-494 promotes cell proliferation, migration and invasion, and increased sorafenib resistance in hepatocellular carcinoma by targeting PTEN

**DOI:** 10.3892/or.2021.8236

**Published:** 2021-11-30

**Authors:** Kai Liu, Songyang Liu, Wei Zhang, Baoxing Jia, Ludong Tan, Zhe Jin, Yahui Liu

Oncol Rep 34: 1003-1010, 2015; DOI: 10.3892/or.2015.4030

Following the publication of this paper, it was drawn to the Editors’ attention by a concerned reader that the western blotting data in Figs. 3 and 6 were strikingly similar to data appearing in different form in other articles by different authors at different research institutes. Owing to the fact that the contentious data in the above article were already under consideration for publication, or had already been published, elsewhere prior to its submission to *Oncology Reports*, the Editor has decided that this paper should be retracted from the Journal. After having been in contact with the authors, they agreed with the decision to retract the paper. The Editor apologizes to the readership for any inconvenience caused.

